# Kidney outcomes are altered by preconception weight modulation in rodent mothers with obesity

**DOI:** 10.1038/s41598-024-68234-9

**Published:** 2024-07-29

**Authors:** Natassia Rodrigo, Hui Chen, Carol A. Pollock, Sarah J. Glastras

**Affiliations:** 1 Renal Research Laboratory, Kolling Institute of Medical Research, St Leonards, NSW Australia; 2 North Precinct, Sydney Medical School, University of Sydney, Sydney, NSW Australia; 3 Department of Diabetes, Endocrinology and Metabolism, Royal North Shore Hospital, Reserve Road, St Leonards, NSW Australia; 4 Department of Diabetes and Endocrinology, Nepean Hospital, Kingswood, NSW Australia; 5 School of Life Sciences, Faculty of Science, University of Technology Sydney, Ultimo, NSW Australia

**Keywords:** Kidney disease, Reproductive health, Liraglutide, Diet, Weight loss, Diabetes, Metabolic syndrome, Obesity, Chronic kidney disease

## Abstract

Obesity increases the risk of chronic kidney disease. We have previously demonstrated the benefits of preconception maternal weight loss on fertility and pregnancy outcomes in a mouse model of maternal obesity. Here, we elucidate if preconception weight loss, either by diet modification or the glucose-like peptide 1 agonist liraglutide, used in the treatment of diabetes and obesity, improves maternal kidney outcomes in late gestation. C57BL/6 female mice were fed either a high-fat-diet (HFD) or a chow (control) diet for 8 weeks. To induce pre-pregnancy weight loss, HFD-fed dams were switched to chow diet (HFD-C) or administered liraglutide (0.3 mg/kg subcutaneous) whilst continuing on HFD (HFD-L). Liraglutide was discontinued one week prior to mating. HFD-V mice continued on HFD, with saline injections. A group of HFD-fed dams were ‘diet switched’ to chow after conception (post-conception, HFD-PC). Maternal body weight and glucose tolerance were measured: (1) preconception and (2) during late gestation followed by blood, urine and kidney collection. Serum creatinine, urinary creatinine and albumin, kidney tissue gene expression and protein were measured. In the preconception period, HFD-L and HFD-C mothers have lower urine albumin:creatinine ratios (UACR) and fatty acid synthase (FAS) protein expression (*P* < 0.005 vs. HFD-V). At late gestation, kidneys of HFD-V and HFD-PC dams have increased gene expression of insulin receptor and FAS (*P* < 0.05) and higher UACR compared to controls (*P* < 0.01). In the HFD-PC group, kidneys show increased mRNA and protein expression of metabolic and oxidative stress markers (FAS, 8-OHdG vs. control, *P* < 0.05, *P* < 0.0001 respectively). The preconception intervention groups with liraglutide, or diet change show reduced oxidative stress (protein expression of 8-OHdG, *P* < 0.05 vs. HFD), mRNA and protein expression of *FAS* (*P* < 0.05 vs. HFD), protein expression of fibrosis markers (collagen IV, fibronectin vs. HFD, *P* < 0.05), and UACR (*P* < 0.05 vs. HFD). This study suggests that preconception weight loss benefits maternal kidney health during pregnancy, superior to diet intervention once already pregnant.

## Introduction

The global pandemic of obesity has far-reaching consequences for women of reproductive age, impacting health outcomes in pregnancy and well beyond the early postpartum period^1^. These include increased risks of gestational diabetes mellitus (GDM) and postpartum type 2 diabetes, hypertension, pre-eclampsia, and long-term risks of cardiovascular and chronic kidney disease (CKD)^2–5^. CKD is a devastating health condition, characterised by structural damage to the renal parenchyma, and progressive loss of renal excretory function^6^. Obesity is an independent risk factor for the development of proteinuria, functional loss reflected by reduced estimated glomerular filtration rate (eGFR) and end stage kidney failure^5^. Concomitant obesity also incites a more rapid decline in kidney function in patients with pre-existing CKD^7^.

The links between CKD, insulin resistance and obesity are well-established^8,9^. Insulin resistance, as seen in obesity^10^, GDM^11^ and type 2 diabetes^12^, is associated with chronic inflammation, through cytokines secreted by adipose resident macrophages^13^, which in turn increases oxidative stress and renal insufficiency^14^. Insulin stimulates insulin-like growth factor 1 production, promoting connective tissue growth factor expression and the development of tubulointerstitial fibrosis in diabetic kidneys^15^. In light of this, targeting obesity and insulin resistance should be a priority in reducing the future risk of CKD in the mother.

Weight loss, facilitated by diet and exercise, has been shown to reduce albuminuria and improve eGFR, thereby improving renal function^16^. GLP-1 agonists (GLP-1RAs) are widely used for both diabetes and obesity management^17^. Liraglutide is a synthetic acylated GLP-1 analog, which acts as a potent agonist at the adenylate cyclase coupled GLP-1 receptor. Following binding, the increase in cyclic adenosine monophosphate (cAMP) stimulates the glucose dependant release of insulin from the pancreas, inhibits glucose dependant release of glucagon, and slows gastric emptying in order to optimise glucose homeostasis^18^. It also acts centrally to reduce food intake and increase satiety^19^. They also exert renal protective effects, through altering renal blood flow and inhibiting inflammation and oxidative stress, attributed to intrinsic properties of the medication and secondary to weight loss itself^20–22^. These effects are seen in both diabetic and non-diabetic models of CKD^21,23^.

Reducing body weight prior to conception is strongly advocated across multiple national guidelines for women with obesity, although the evidence of benefit remains equivocal^24^. Deferring pregnancy until optimal body weight is achieved is difficult as pregnancy and windows of fertility are time sensitive^25^. We have shown that pre-pregnancy administration of the GLP-1RA, liraglutide, can facilitate weight loss and improve fertility in a mouse model of maternal obesity^26^. To date, no studies have investigated if pre-pregnancy weight loss using GLP-1RA, or dietary intervention before or during pregnancy, has beneficial effects on maternal kidney outcomes in the setting of maternal obesity. Such findings would have significant clinical value, as it has the potential to alter the risk of cardiorenal complications in pregnancy, and CKD in women of reproductive age.

Therefore, this study aimed to determine if pre-pregnancy diet modification, or liraglutide treatment, can improve preconception and late gestation kidney health in a mouse model of maternal obesity. We hypothesised that pre-pregnancy weight loss, especially facilitated by the potent GLP-1RA liraglutide, would improve kidney outcomes, and specifically reduce renal inflammation and oxidative stress within the kidneys of obese dams. We also hypothesised that pre-pregnancy weight loss would be more effective at protecting maternal kidney health, compared to weight intervention strategies initiated during pregnancy.

## Results

### Preconception kidney outcomes

1. Kidney: body weight ratios in the preconception period

The kidney: body weight ratios were significantly lower in the HFD-V group compared to controls in the preconception period (*P* < 0.01, Table 1), driven by changes in body weight, not kidney weight, as previously described^26^. The HFD-L group had a greater kidney: body weight ratio, compared to HFD-V (left and right, both *P* < 0.001). Similarly, the HFD-C group had a higher kidney to body weight ratio, compared to HFD-V (left, *P* < 0.05 right, *P* < 0.05). There was no difference in kidney size between the control, HFD-L and HFD-C groups.

**Table 1:** Kidney: body weight, expressed as a percentage, in preconception and late gestation dams.

%Kidney: body weight		C	HFD-V	HFD-L	HFD-C	HFD-PC	*P* value
Preconception	Left	0.75 ± 0.03	0.58 ± 0.03	0.78 ± 0.05	0.78 ± 0.03		0.003
	Right	0.75 ± 0.03	0.59 ± 0.02	0.79 ± 0.05	0.79 ± 0.03		0.002
Late gestation	Left	0.81 ± 0.1	0.62 ± 0.02	0.64 ± 0.03	0.89 ± 0.11	0.67 ± 0.04	0.01
	Right	0.81 ± 0.1	0.62 ± 0.92	0.64 ± 0.03	0.89 ± 0.11	0.67 ± 0.04	0.01

Results expressed as mean ± SEM, N = 8 per preconception group, N = 12 per late gestation group. *P* value for ANOVA.

2. Kidney function in the preconception period

UACR was significantly higher in the HFD-V group compared to the control group (*P* < 0.0005, Table 2). The HFD-L group had significantly reduced UACR compared to HFD-V (*P* < 0.01), although still twice the level of the control group (*P* < 0.05). The HFD-C group had a reduced UACR compared to the HFD-V group (*P* < 0.005). There was no significant difference in UACR between HFD-L or HFD-C groups or between HFD-C and control groups (Table 2).

**Table 2:** Preconception and late gestation urine and serum markers.

Renal function		C	HFD-V	HFD-L	HFD-C	HFD-PC	*P* value
Pre-conception	Urine Albumin: creatinine ratio (µg/ml)	25.73 ± 8.1	110.0 ± 19.8	58.22 ± 8.4	31.9 ± 6.3		0.0007
	Serum creatinine (µmol/L)	28.97 ± 0.7	27.58 ± 0.66	27.82 ± 1.01	28.27 ± 0.56		NS
Late gestation	Urine Albumin: creatinine ratio (µg/ml)	10.66 ± 1.2	81.67 ± 34.0	30.30 ± 10.0	11.57 ± 2.0	27.36 ± 7.9	0.04
	Serum creatinine (µmol/L)	29.36 ± 0.9	30.08 ± 0.9	29.85 ± 0.4	28.57 ± 0.6	28.65 ± 0.7	NS

Results expressed as mean ± SEM, N = 8 per group. *P* value for ANOVA.

There were no significant differences in serum creatinine between the groups (Table 2).

3. Renal oxidative stress markers in the preconception period

**Figure 1:**
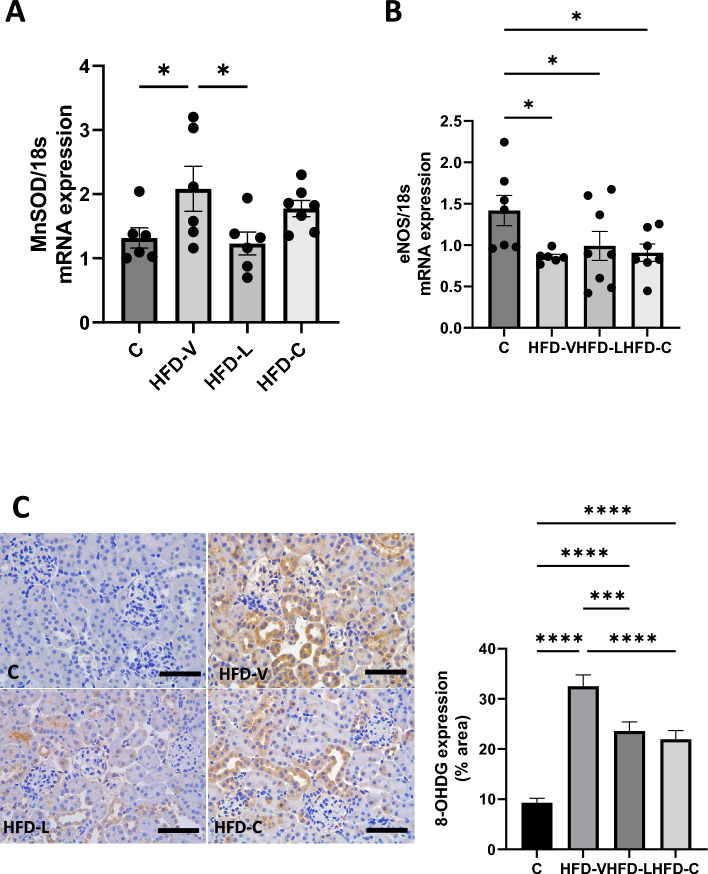
Renal oxidative stress markers in the preconception period. (**A**) MnSOD mRNA expression, relative to 18S. (**B**) eNOS mRNA expression, relative to 18S. (**C**) 8-OHDG protein expression by immunohistochemistry. N = 8 per group, results expressed as mean ± SEM, *P < 0.05, **P < 0.005 ***P < 0.0005 ****P < 0.0001. *P* value for ANOVA. (scale bar = 100 μm).

The mRNA expression of *MnSOD*, an endogenous enzyme to scavenge free radicals^27^, was significantly higher in the HFD-V group compared to controls (*P* < 0.05, Fig. 1A). HFD-L mice had lower *MnSOD* mRNA levels than HFD-V (*P* < 0.05), similar to the control level. The *MnSOD* mRNA levels in the HFD-C group did not differ from the other groups.

The mRNA expression of the oxidative stress marker, *eNOS*, was reduced in the HFD-V, HFD-L and HFD-C groups versus C (all *P* < 0.05, Fig. 1B). Similarly, *8-OHdG* protein, a marker of oxidative stress-induced DNA damage, was significantly higher in the HFD-V group (*P* < 0.0001 vs control), which was significantly reduced in the HFD-L and HFD-C groups (*P* < 0.0005 HFD-L and HFD-C vs HFD-V; *P* < 0.0001), but still significantly higher than the control group (*P* < 0.0001 HFD-L and HFD-C vs control, Fig. 1C).

There was no difference in inflammatory markers, IL-6mRNA and CD-68mRNA, between groups. Protein markers of inflammation were therefore not pursued.

4. Renal metabolic markers in the preconception period

**Figure 2:**
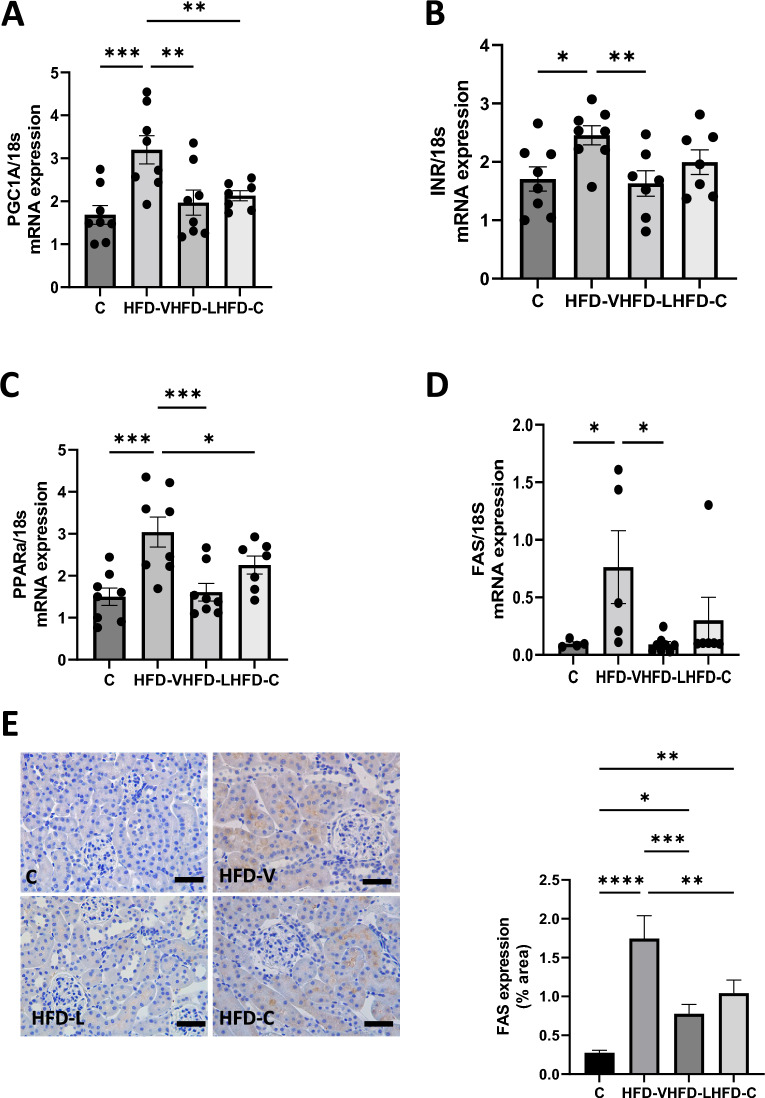
Renal metabolic markers in the preconception period. (**A**) PGC1α mRNA expression, relative to 18S. (**B**) Insulin Receptor mRNA expression, relative to 18S. (**C**) PPARα mRNA expression, relative to 18S. (**D**) FAS mRNA expression, relative to 18S. E. FAS protein expression by immunohistochemistry. N = 8 per group, results expressed as mean ± SEM, *P < 0.05, **P < 0.005 ***P < 0.0005 ****P < 0.0001. *P* value for ANOVA. (scale bar = 100 μm).

mRNA expression of *PGC1α*, involved in mitochondrial biogenesis and cellular energy metabolism^28^, was significantly higher in the HFD-V group (*P* < 0.0005 vs control, Fig. 2A), which was reduced in the HFD-L (*P* < 0.005 vs HFD-V) and HFD-C (*P* < 0.01).

*InR* mRNA expression was significantly higher in the HFD-V compared to the control group (*P* < 0.05, Fig. 2B), which was reduced in the HFD-L group (*P* < 0.01 vs HFD-V). *InR* mRNA expression was not altered in the HFD-C group.

*PPARα* mRNA expression, a transcription factor involved in fatty acid metabolism and anti-inflammatory activity in the kidney^29,30^, was significantly higher in the HFD-V group (*P* < 0.0005 vs control, Fig. 2C). It was significantly lower in the HFD-L group (*P* < 0.001 vs HFD-V). *PPARα* in the HFD-C group was also significantly lower than the HFD-V group (*P* < 0.05).

Similarly, *FAS* mRNA expression was significantly higher in the HFD-V group (*P* < 0.05 vs control, Fig. 2D), and reduced in the HFD-L group (*P* < 0.05 vs HFD-V). *FAS* mRNA expression in the HFD-C group was not significantly different from the other groups. *FAS* protein level in the HFD-V group mirrored mRNA expression (*P* < 0.0001 vs control, Fig. 2E). Protein levels in both HFD-L and HFD-C groups were significantly lower than in the HFD-V groups (*P* < 0.0005, *P* < 0.005, respectively). However, they were not different from the control group.

5. Renal fibrotic markers in the preconception period

**Figure 3:**
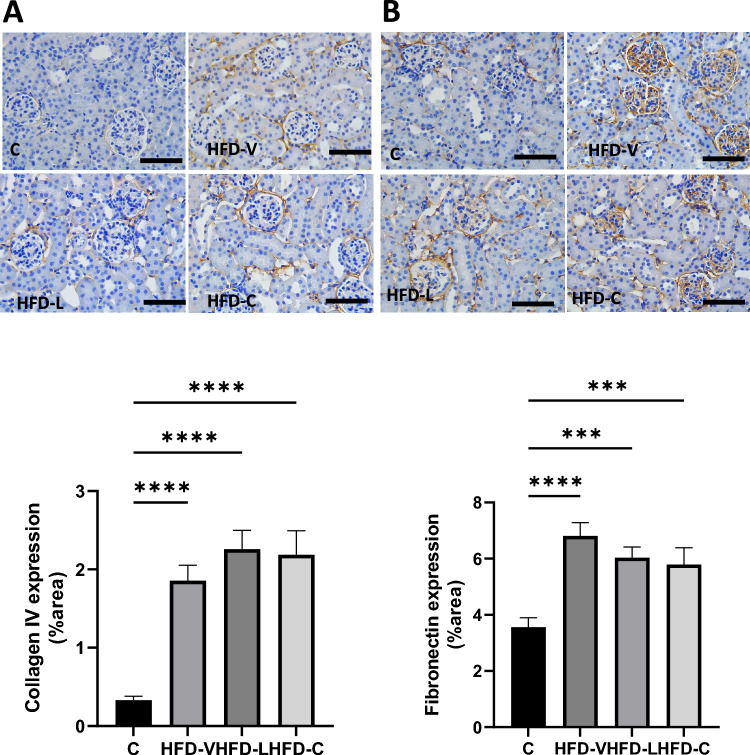
Renal fibrosis markers in the preconception period. (**A**) Collagen IV protein expression by immunohistochemistry. (**B**) Fibrinogen protein expression by immunohistochemistry. N = 8 per group, results expressed as mean ± SEM, *P < 0.05, **P < 0.005 ***P < 0.0005 ****P < 0.0001. *P* value for ANOVA. (scale bar = 100 μm).

Col IV protein expression was significantly increased in the HFD-V, HFD-L, and HFD-C groups to a similar level (all *P* < 0.0001 compared to the control, Fig. 3A). FN protein was significantly increased in the HFD-V mice (*P* < 0.0001 compared to control), but to a lesser extent in the HFD-L (*P* < 0.0005 vs control) and HFD-C groups (*P* < 0.001 vs control, Fig. 3B).

### Late gestation kidney outcomes

1. Kidney: body weight ratios in late gestation

At late gestation, the kidney: body weight ratios were similar between HFD-V, HFD-L and control groups (Table 1). The kidney: body weight ratios of the HFD-L and HFD-V groups were significantly smaller than the HFD-C group (*P* < 0.005). The kidney: body weight ratio of the HFD-PC group was also significantly smaller than the HFD-C group (*P* < 0.05), again due to greater body weight rather than smaller kidney sizes, as previously described^26^.

2. Kidney function in late gestation

UACR was the highest in the HFD-V group (*P* < 0.05 vs control, HFD-L, and HFC-PC; *P* < 0.01 vs HFD-C Table 2). There was no difference in UACR among the control, HFD-L, HFD-C and HFD-PC groups (Table 2). There were no significant differences in serum creatinine levels between the groups (Table 2).

3. Renal oxidative stress makers in late gestation

**Figure 4:**
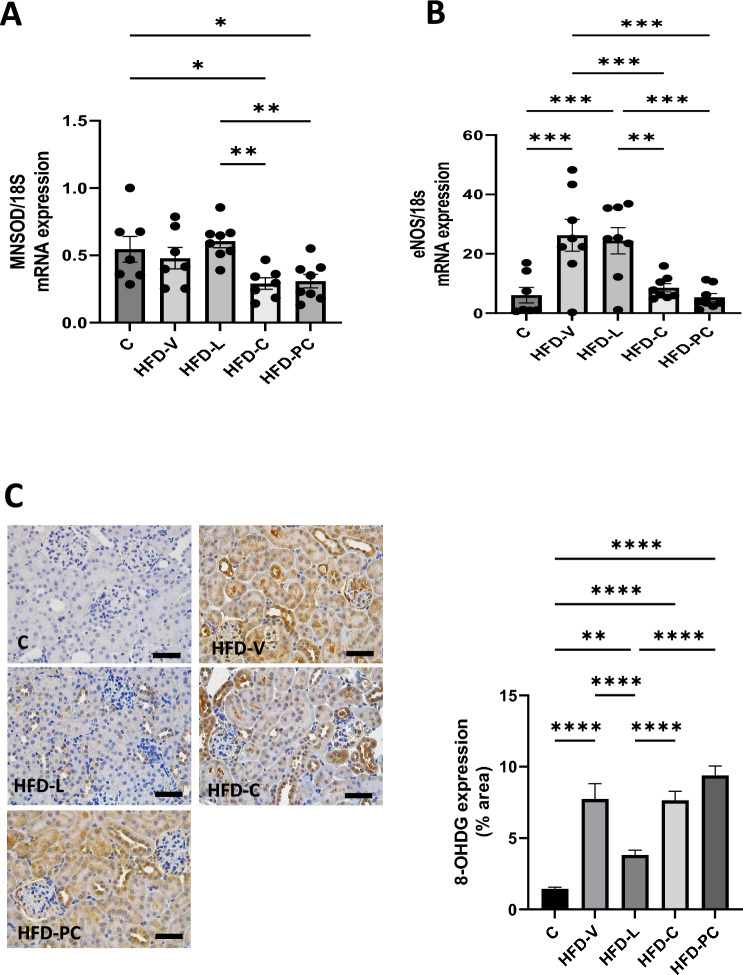
Renal oxidative stress makers in late gestation. (**A**) MnSOD mRNA expression, relative to 18S. (**B**) eNOS mRNA expression, relative to 18S. (**C**) 8-OHDG protein expression by immunohistochemistry. N = 8 per group, results expressed as mean ± SEM, *P < 0.05, **P < 0.005 ***P < 0.0005 ****P < 0.0001. *P* value for ANOVA. (scale bar = 100 μm).

At late pregnancy, *MnSOD* mRNA kidney expression was not significantly different between the HFD-V and control groups (Fig. 4A). *MnSOD* mRNA expression was significantly lower in the HFD-C and HFD-PC group than in the controls (both *P* < 0.05) and the HFD-L group (both *P* < 0.005). There was no difference in *MnSOD* mRNA expression between the control, HFD-L or HFD-V groups.

*eNOS* mRNA renal expression was significantly higher in the HFD-V and HFD-L groups than in the controls (both *P* < 0.0005 Fig. 4B). *eNOS* mRNA levels in the HFD-Cand HFD-PC groups were similar to controls.

*8-OHdG* protein expression was significantly increased in the HFD-V, HFD-C and HFD-PC groups to a similar level (all *P* < 0.0001 vs control, Fig. 4C). Furthermore, *8-OHdG* in the HFD-L group was significantly lower than the HFD-V group (*P* < 0.01).

There was no difference in inflammatory markers, IL-6mRNA and CD-68mRNA, between groups. Protein markers of inflammation were therefore not pursued.

4. Renal metabolic markers in late gestation

**Figure 5:**
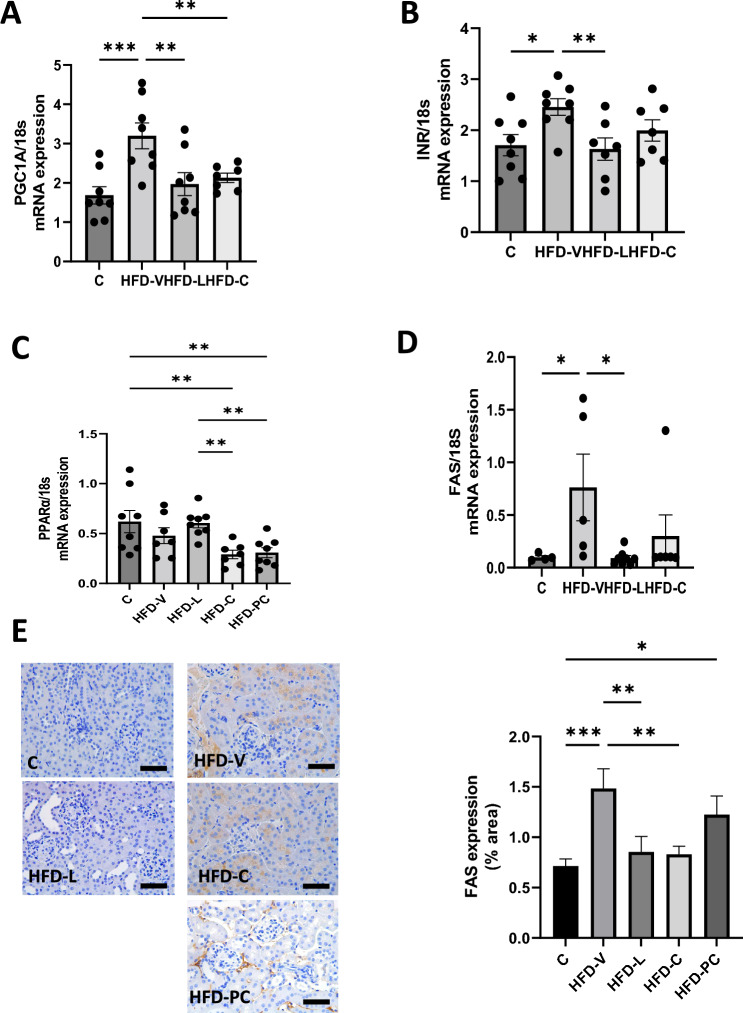
Renal metabolic markers in late gestation. (**A**) PGC1α mRNA expression, relative to 18S. (**B**) Insulin Receptor mRNA expression, relative to 18S. C. PPARα mRNA expression, relative to 18S. (**D**) FAS mRNA expression, relative to 18S. (**E**) FAS protein expression by immunohistochemistry. N = 8 per group, results expressed as mean ± SEM, *P < 0.05, **P < 0.005 ***P < 0.0005 ****P < 0.0001. *P* value for ANOVA. (scale bar = 100 μm).

*PGC1-α* mRNA expression was not significantly different between the control and HFD-V groups but increased in the HFD-L group compared to the control group (*P* < 0.005, Fig. 5A). However, *PGC1-α* mRNA expression in the HFD-C and HFD-PC groups was similar to the control group, and lower than the HFD-V group (*P* < 0.005, *P* < 0.0005 respectively).

*InR* mRNA expression was not significantly different between the control and other treatment groups (Fig. 5B). However, the levels in the HFD-C and HFD-PC groups were lower than the HFD-V group (both *P* < 0.01).

*PPAR α* mRNA expression was not significantly different between control, HFD-V and HFD-L groups (Fig. 5C). It was significantly lower in HFD-C and HFD-PC groups compared to the control group (both *P* < 0.01).

*FAS* mRNA expression was not significantly different between the control, HFD-V and HFD-L groups but was lower in the HFD-C and HFD-PC groups compared to HFD-V(both *P* < 0.005, Fig. 5D). However, FAS protein expression was significantly higher in the HFD-V and HFD-PC groups compared to the control group (*P* < 0.001, *P* < 0.05, respectively), but reduced in the HFD-L and HFD-C groups (both *P* < 0.01 vs HFD-V, Fig. 5F).

5. Renal fibrotic markers in late gestation

**Figure 6:**
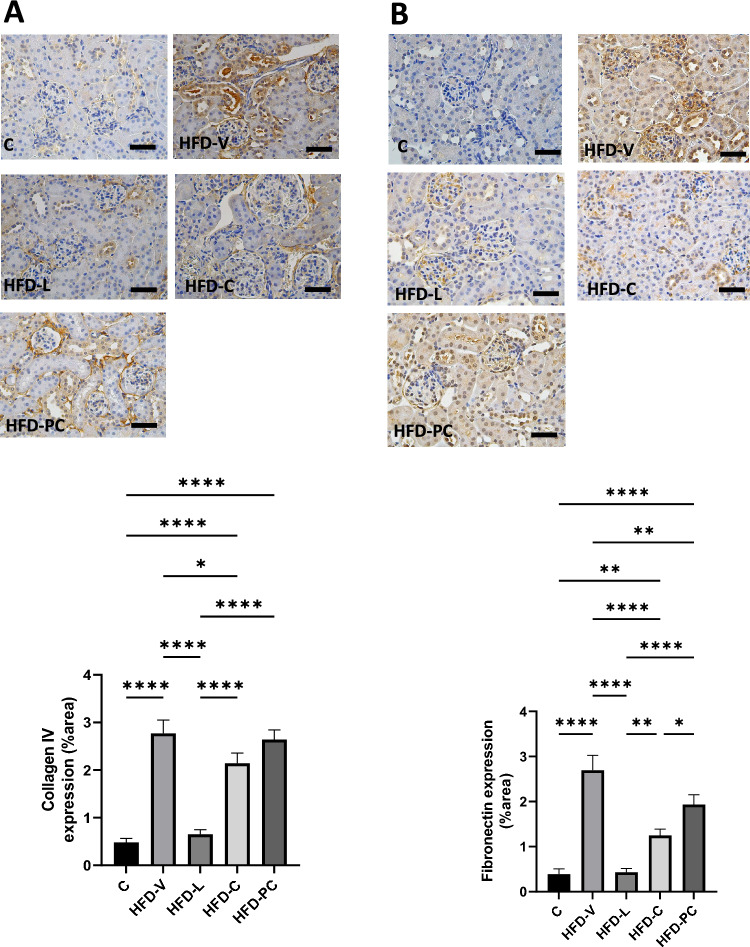
Renal fibrosis markers in late gestation. (**A**) Collagen IV protein expression by immunohistochemistry. (**B**) Fibrinogen protein expression by immunohistochemistry. N = 8 per group, results expressed as mean ± SEM, *P < 0.05, **P < 0.005 ***P < 0.0005 ****P < 0.0001. *P* value for ANOVA. (scale bar = 100 μm).

Col IV protein expression was significantly higher in HFD-V compared to control (*P* < 0.0001). It was reduced in the HFD-L group (*P* < 0.0001 vs HFD-V), but not in the HFD-C (*P* < 0.05 vs HFD-V, *P* < 0.0001 vs control) and HFD-PC groups (*P* < 0.0001 vs control, Fig. 6A).

FN protein expression was also significantly higher in HFD-V group compared to control group (*P* < 0.0001), which was reduced in the HFD-L group (*P* < 0.001 vs HFD-V). The HFD-C group also had reduced FN protein expression compared to the HFD-V group (*P* < 0.0001), although less marked than the HFD-L group (*P* < 0.005). The HFD-PC group also showed some benefits compared to HFD-V (*P* < 0.01, Fig. 6B).

## Discussion

This novel study, investigating the kidney benefits of weight modulation prior to pregnancy, found that preconception weight reduction with liraglutide yielded maternal kidney benefits in the preconception period sustained into late pregnancy despite ongoing maternal HFD. At both timepoints, a reduction in albuminuria, oxidative stress markers (MnSOD and 8-OHdG) and metabolic markers (PPARα, InR, and FAS) were observed. Preconception dietary modification also had benefits on renal outcomes, both in the preconception and at late gestation, with reduced albuminuria, oxidative stress markers (8-OHdG, MnSOD and eNOS) and metabolic markers (PGC1α and PPARα). As compared with dietary intervention, pre-pregnancy liraglutide significantly reduced late gestation kidney fibrotic markers (Col IV and FN), demonstrating the protective potential of GLP-1RA on metabolic end organ damage.

Preconception maternal obesity has known effects on the kidney in pregnancy with an increased risk of proteinuria, gestational hypertension, and pre-eclampsia^31–33^. There is also ongoing health sequalae for the mother beyond the pregnancy itself, with risks of metabolic disease including chronic hypertension, diabetes mellitus, ischaemic heart disease and kidney disease^33,34^. Our study provides further evidence of the deleterious impact of maternal obesity on the maternal kidney. The mechanisms underlying this include increased inflammation and oxidative stress which are exaggerated in pregnancy, leading to increased fibrotic changes with abnormal lipid metabolism^35^. These same markers of pathophysiologic change are recapitulated in our study. Albuminuria was higher in the maternal HFD-fed group at both time points, reflective of known effects of obesity on kidney function^36^. Further, renal oxidative stress, metabolic and fibrotic markers were elevated in the HFD-V group in the preconception period, a detrimental pattern common to both CKD and obesity, which continued into late gestation^37^. FAS and InR levels were predictably higher in the HFD-V mice at both time points studied, as both are upregulated in obesity^35,38,39^. PPARα, which stimulates the uptake and oxidation of fatty acids, reflecting upregulated nutrient metabolism, was elevated in the preconception period in HFD-V mice. Activation of PPARα has been seen in obese states as a counteractive mechanism, where PPAR responsive genes involved in lipid transport are upregulated, leading to increased fat storage^40^. PGC1α was consistently elevated in HFD-V, overexpression of which is seen in obesity, with altered β-cell insulin function and apoptosis, and activation of glucose transporter 4^41^. Interestingly, eNOS mRNA expression was higher in the control group in the preconception period, compared to the HFD-fed mice. This is a known phenomenon seen in obesity and HFD feeding, in which there is suppression of eNOS production through down regulation of the AMP-activated protein kinase pathway, correlating with serum TAG levels and glucose tolerance profiles, as a means of modulating energy storage^42,43^.

This study elucidates that weight loss in the preconception period, either by diet or liraglutide, protects the HFD-fed dam from albuminuria, and metabolic and oxidative stress prior to commencement of pregnancy. Both modes of pre-pregnancy weight loss led to reduced 8-OHdG, PPARα, PGC1α, and FAS expression compared to obese mice. Downregulation of these markers suggests enhancement of mitochondrial biogenesis, suppression of inflammation and mitigation of oxidative stress^41,44–46^. Mitochondrial biogenesis, where cells increase mitochondrial mass in response to cellular stress, is upregulated in obesity, due to its role in fatty acid oxidation, with dysfunction in the face of adipokine secretion leading to defects in lipid and glucose homeostasis^47^. Interestingly, liraglutide, despite ongoing HFD, facilitated the most pre-pregnancy weight loss, reduced de novo fatty acid synthesis through reduced FAS activity and improved metabolic and oxidative stress to a greater extent than diet change alone. This is in keeping with the known mechanism of action of GLP-1 receptor agonists which facilitate potent weight loss and metabolic optimisation^48^. MnSOD mRNA expression, reduced in the HFD-L group, is known to be down regulated with GLP-1RA therapy, and may reflect a reduced requirement of antioxidant activity from liraglutide^49^. InR expression was lowest in the HFD-L, but not HFD-C group, most likely because of the known greater efficacy GLP-1RAs have on insulin receptor activity, glucose uptake and insulin resistance^50^. Interestingly, neither method of preconception weight loss made any appreciable difference to preconception fibrotic markers in the kidney. This may reflect the short time frame between therapy and measurement, especially in light of the significant reduced renal fibrosis appreciated by late gestation.

The mechanisms of GLP-1 receptor agonists and their effects across metabolic, inflammatory and oxidative stress pathways have been widely studied^51^. Similar effects to our study have been demonstrated with Exendin 4, another GLP-1RA, which was administered to non-pregnant rats whilst consuming HFD and showed significant benefits in renal oxidative stress and lipid accumulation^20^ . GLP-1 receptors, while predominantly expressed in β-cells of the pancreas, are ubiquitously expressed in various tissues throughout the body including the renal vasculature, allowing for direct kidney based GLP-1 activation and action^52^. They also act indirectly by interacting with the renin–angiotensin–aldosterone system, by attenuation of the angiotensin II response, leading to reduced intra-renal hypertension^53^. These are known pathogenic pathways in the development of nephropathy associated with diabetes and obesity^5,54^. Liraglutide has been shown to inhibit glomerular superoxide and NADPH, thereby alleviating diabetic nephropathy through protection against renal oxidative stress^55^. Exendin 4 has also been shown to upregulate ATP-binding cassette transporter A1 expression, which promotes cholesterol efflux from cells and inhibits inflammatory responses, reducing renal lipid synthesis, inflammation, and proteinuria^55^. Interestingly, metabolomic studies using liraglutide show reduced renal lipids, including fatty acid residues, cholesterol, and TAG, with improvements in mitochondria metabolites such as succinate, citrate and taurine, rebalancing renal metabolism in obesity related kidney disease^56^. Such findings add mechanistic weight for adjunctive use of GLP-1RAs in the pre-pregnancy state, over diet modification alone, as a weight modulator in the context of maternal obesity, as it offers pleiotropic effects beyond weight loss. This is supported by our study, which demonstrates, at least in the context of gestational weight gain (GWG), improved renal function in the group treated with liraglutide. Further, the sustained benefit, despite the need to cease GLP-1RAs prior to conception, due to their potential teratogenicity^57^, is promising.

We previously reported that the maternal HFD-fed group treated with liraglutide in the preconception period had significant GWG^26^. Nonetheless, there was a protective ‘legacy effect’ from the prior weight loss with liraglutide, such that renal functional and structural changes, as measured by albuminuria, renal fibrosis-related changes (Col IV and FN) and oxidative DNA damage (8-OHdG) were lower at late gestation, despite the accelerated weight gain in pregnancy. Acute markers of oxidative stress (MnSOD and eNOS) were not significantly different between the HFD-L and HFD-V groups, most likely indicating that the metabolic consequences of GWG was affecting ‘short term’ acute antioxidant mechanisms by late pregnancy. The concept of a ‘legacy effect’ is not novel in the area of metabolic disease, with compelling evidence arising from the Epidemiology of Diabetes Interventions and Complications (EDIC) study, UK Prospective Diabetes Study (UKPDS) and Steno-2 trial showing that newly diagnosed patients with either type 1 or type 2 diabetes, who achieve early tight glycaemic control, have lower long-term risk of microvascular complications including diabetic nephropathy and progression of CKD^58–60^. Importantly, these effects were seen despite a relaxation of glycaemic control over time, demonstrating that a period of early good control can exert enduring benefit. The short-term protective benefit of weight loss through intermittent fasting on dyslipidaemia, hypertension and inflammation has also been demonstrated, with improvements seen within the first month of diet change, and temperance of benefit seen weeks after the resumption of normal diet^61,62^. Our study provides further evidence that short-term changes in body weight can be beneficial for kidney health in pregnancy.

Our study found that diet switch in pregnancy led to mixed renal benefits, with kidney function (albuminuria) similar, compared to lean controls, but evidence of early fibrosis with elevated Col IV protein expression. Reduced expression of oxidative stress markers, such as MnSOD and eNOS, but not 8-OHdG expression, reflected similar benefits seen in the HFD-C mice. These partial improvements are likely to reflect the short time span between the intervention and measurement of these markers. Mice who underwent the diet switch in pregnancy had a longer unopposed exposure to HFD, and its negative impacts on the kidney, especially as relates to chronic changes such as developing fibrosis. It can be conjectured that, following diet change to a less obesogenic diet, acute phase reactants, such as oxidative stress markers, as measured by mRNA, are more likely to be initially regularised. Following these mice longer term, past the pregnancy, would be of interest, to see if such changes manifest in reduced fibrosis compared to mice continuously on HFD.

This constitutes one limitation of this study. Kidney disease, both in obese mice models, and in humans’ pathophysiology, constitutes a long-term consequence of chronic exposure to this detrimental milieu. Changes seen in mice of 12–16 weeks of age are early at best. Longer term analysis of older age mice, with these experimental exposures, would be beneficial to greater explore the effect of obesity, pregnancy and preconception interventions on long term kidney health. This was not possible in the study design of the current study, in which mice were terminated in late gestation. Moreover, pregnancy is a period of rapid physiological change, with increased inflammatory markers and oxidative stress generally during pregnancy and peaking close to delivery. Mice gestation is short, at only 21 days in total. Exploration of inflammatory pathways showed no difference at the time of harvest, which given its temporal proximity to natural labour in our mice, is, in retrospect, not surprising. Further protein analysis to explore this was therefore not undertaken. A further constraint of this study is the incapacity to differentiate between GLP-1RA effects of weight loss, glucose optimisation, and direct targeting of the kidney parenchyma. This however, may be considered of less clinical significance as these effects are concomitant in the human experience. The use of a HFD feeding model of obesity to mimic maternal overweight/obese conditions is a strength of this study, given its capacity for human correlation. The exploration of weight loss by two modalities, diet and liraglutide^26^, provides an excellent model with which to examine renal outcomes, given the topical nature of these drugs and the metier in the fields of type 2 diabetes and obesity^17^. The exploration of renal maternal outcomes is useful clinically.

Preconception weight loss reduced albuminuria and oxidative stress markers both in the preconception period and favourably benefited weight, glucose tolerance and renal outcomes in late pregnancy. Most interestingly, pre-pregnancy liraglutide facilitated kidney protection, despite accelerated GWG and reduced glucose tolerance in late pregnancy. Our study therefore suggests that preconception use of GLP-1 receptor agonists has maternal renal benefits in mothers with obesity. While further studies in human populations are required to confirm maternal and neonatal protection from pre-pregnancy weight loss intervention, our findings provide promise of metabolic and renal benefit of pre-conception GLP1-RA intervention for reproductive-age females with obesity.

## Methods

### Mouse model

4-week-old, female C57Bl/6 mice were obtained (Kearns Facility, Kolling Institute, St Leonards, NSW, Australia, N = 96). To allow for socialisation, mice were housed in groups of 3–4 per cage, with ad libitum access to food and water, maintained at 22 ± 1°C with a 12-h light–dark cycle, humidity between 40–60%. Weekly animal monitoring, including weight, was maintained with all procedures approved by the Animal Care and Ethics Committee (AEC) of the Northern Sydney Local Health District (RESP/18/148) with compliance according to the Australian Code of Practice for the Care and Use of Animals for Scientific Purposes as well as the Animal Research: Reporting of In Vivo Experiments (ARRIVE) Guidelines.

Mice were randomly allocated into 2 groups, in a ratio of 1:3, to be fed either a standard chow diet (11kJ/g, 14% fat and 21% protein) or a high fat diet (HFD comprising 20kJ/g, 43% fat and 21% protein; SF04-001; Specialty Feeds, WA, Australia) for 8 weeks, to induce obesity^63^. Sample sizes were determined based on previous work^63^. All mice in a single cage underwent the same grouping and subsequent treatment procedure. Stratified randomisation using body weight was used in order to reduce bias. Afterward, mice underwent 4 weeks of weight loss intermediation, in what is termed the preconception period. To do this, HFD-fed mice were again randomly divided into 4 groups, in a 1:1:1:1 ratio: (1) HFD-V: HFD-vehicle (2) HFD-L: HFD in combination with liraglutide, (3) HFD-C: HFD switched to chow in the preconception period, (4) HFD-PC: HFD switched to chow only once pregnancy was confirmed (termed post-conception). Chow fed mice (controls) continued on chow diet throughout the preconception and pregnancy periods. The HFD-V group were continued on HFD throughout this preconception period and during pregnancy. The HFD-C group underwent diet change from HFD to chow, initiated in this 4-week period and maintained thereafter during mating and pregnancy. The HFD-PC group continued on HFD, until pregnancy was confirmed, at which point they were switched to a chow diet. The HFD-L group underwent daily liraglutide subcutaneous injections, in conjunction with a HFD, which was maintained throughout the preconception and pregnancy periods. In order to reduce adverse effects of liraglutide, such as nausea and gastrointestinal discomfort, dose escalation was undertaken every 3 days, with a starting dose of 0.1mg/kg/day, followed by 3 days of 0.2mg/kg/day, to a full treatment dose of 0.3mg/kg/day, as previously described^64^. All other groups, including the control group, received subcutaneous saline injections with the same volume incrementation as liraglutide therapy. Mice were weighed at the end of this 4 week treatment period. A group of mice (N = 8 per group, C, HFD-V, HFD-L, HFD-C) were sacrificed at this point, with kidneys harvested and weighed, and blood and urine collected at preconception. At the time of cull, mice were allocated a study code, to blind researchers to the treatment group for all further analyses, including tissue and bioassay analyses. The ratio of kidney to body weight was calculated and expressed as a percentage of total body weight. Culling procedure involved anesthetisation with isoflurane. Cardiac puncture was performed to obtain blood samples and to euthanise the mice. Following this, kidneys were harvested. Blood was immediately centrifuged and serum was separated and stored at − 80°C for later analysis. Urine was stored at − 30°C for later analysis. Immediately on harvest, kidneys were immersed in liquid nitrogen and subsequently stored at − 80°C for later analysis.

In the remainder of the mice (N = -12 per group, C, HFD-V, HFD-L, HFD-C, HFD-PC), following a one week wash out period, intended to minimise the risk of teratogenic effects of liraglutide, dams underwent male co-housing (male to female ratio 1:3) for 3 days. If pregnancy was not achieved, 2 further cycles of 3 days of mating were attempted. A priori, mice that did not fall pregnant were excluded. The presence of a vaginal plug and weight gain indicated pregnancy. Once pregnancy was confirmed, pregnant female mice were housed individually until the end of gestation.

At day 18 of gestation, pregnant mice underwent a intraperitoneal glucose tolerance test (IPGTT) to assess glucose tolerance in late gestation, the methodology of which has been previously described^26^. Researchers were blinded to the treatment group at the time of IPGTT and mice underwent IPGTT in random order to again reduce bias. Briefly, mice were weighed on the test day, fasted for a period of 6 h, 50% glucose (2g/Kg) was administered at time 0min via intraperitoneal injection, and tail tip blood glucose levels were measured at 0 min, 15 min, 30 min, 60 min, 90 min and 120 min using a Roche AccuCheck Performa Meter. Subsequent to the IPGTT, on gestational day 19–20, following a 4-h fast, pregnant dams were sacrificed, in the method described above, with kidney, blood and urine harvested and stored.

### Bioassays

Serum creatinine was measured using the Architect C16000 Clinical Chemical Analyzer (Abbott Laboratories, Abbott Park, IL, USA). Urine Creatinine was determined using a colorimetric assay kit (Cayman Chemical, Michigan, USA) and urine albumin was measured using ELISA (Crystal Chem, IL, USA). The urinary albumin: creatinine ratio (UACR) was then calculated.

### Real time (RT) PCR

RNA was extracted using the RNeasy Plus Mini Kit (Qiagen, CA, USA), and purified total RNA was used as a template to generate cDNA using the iScript cDNA Synthesis Kit (Bio-Rad, CA, USA). RT-PCR was performed using the QuantiNova PCR kit (Qiagen, Hilden, Germany). Oxidative stress markers measured included mitochondrial antioxidant manganese superoxide dismutase (MnSOD) and endothelial nitric oxide synthase(eNOS). Metabolic markers measured included peroxisome proliferator-activated receptor gamma co-activator 1-α (PGC1α), Insulin receptor (InR), Peroxisome proliferator-activated receptor α (PPARα) and fatty acid synthase (FAS). Mouse primers are listed in Table 3. RT-PCR was carried out with the QuantStudio 12 K Flex Real-Time PCR System (Thermo Fisher Scientific). The cycle threshold (Ct) value was analysed using the delta-delta-Ct method. Results were normalised to 18S and expressed as fold change.

**Table 3:** Mouse specific primers used in quantitative real time PCR.

Gene	Forward primer sequence	Reverse primer sequence
18S	ACCGCAGCTAGGAATAATGGA	GCCTCAGTTCCGAAAACC
MnSOD	CACTCTAAGAAACATGG	GATCACACGATCTTCAATGG
PGC1 α	CTCTCAGTAAGGGGCTGGTT	ATCCACTCTGACACACAC
FAS	TGCTCCCAGCTGCAGGC	GCCCGGTAGCTCTGGGTGA
InR	CTGGGAGTGGAGCAAACACAAAC	TGGTCTTCATGGGCAATGTCG
Enos	CCTGGAGTAAAGAACTGGGAAGTG	AACTTCCTGGAAACACCAGGG
PPARα	GGGCTCTCCCACATCCTT	TGGTCTTCAGGGCAATGTCG

### Immunohistochemistry

Formalin-fixed kidneys were sectioned (4 µm) and placed on slides, which were deparaffinised with xylene, rehydrated in graded concentrations of ethanol and rinsed in water. They subsequently underwent heat retrieval in a water bath at 99 °C for 20 min using 0.01 M citrate buffer, pH 6. Slides were then cooled at room temperature for 20 min, rinsed with water and washed in Tris-buffered saline (50 mM Tris, 150 mM NaCl, 0.05% Tween-20, pH 7.6, TBST). Endogenous peroxidase was quenched using 0.3% hydrogen peroxide (Sigma-Aldrich, Dublin, Ireland). Slides were blocked for 10 min with Protein Block Serum-Free (Dako, Glostrup, Denmark), and incubated at 4 °C overnight with the following primary antibodies: fibronectin (FN) (dilution 1:1000, Abcam, Cambridge, UK, catalogue #2413), collagen IV (Col IV) (1:1000, Abcam, catalogue #6586), 8-hydroxy-2’-deoxyguanosine (8-OHdG) (1:1000, Bioss, MA, USA, catalogue #BS-1278R), fatty acid synthase (1:100, Cell Signal, #3180S). Slides were washed with TBST, then incubated with horseradish peroxidase anti-rabbit Envision system (Dako, Japan), and stained for 10 min with 3,3’-diaminobenzidine tetrahydrochloride and counterstained with Mayer’s haematoxylin (Sigma-Aldrich, Sydney, Australia), followed by Scott’s solution. Slides were then rinsed in water, dehydrated using graded concentrations of ethanol and xylene before coverslips were mounted. A digital camera attached to a microscope captured 4–6 non-overlapping images at 40 × magnification, prior to Image J software being utilised to objectively quantitate the area stained.

### Statistical methods

All results are expressed as mean ± standard error of the mean (SEM). Data were analysed using one-way ANOVA, with post hoc Tukey’s tests performed to determine significance (GraphPad Prism 9.0, GraphPad Software, San Diego, CA, USA). The trapezoidal rule was used to determine the area under the curve (AUC) for IPGTT results. *P* < 0.05 was considered statistically significant.

## Data Availability

All data generated or analysed during this study are included in this published article. The datasets generated during and/or analysed during the current study are available from the corresponding author on reasonable request.

## References

[1] Silvestris, E. *et al.* Obesity as disruptor of the female fertility. *Reprod. Biol. Endocrinol. RB&E* **16**(1), 22–22 (2018). 29523133 10.1186/s12958-018-0336-z PMC5845358

[2] Knight, M. *et al.* Extreme obesity in pregnancy in the United Kingdom. *Obstet. Gynecol.* **115**(5), 989–997 (2010). 20410773 10.1097/AOG.0b013e3181da8f09

[3] Catalano, P. M. The impact of gestational diabetes and maternal obesity on the mother and her offspring. *J. Dev. Orig. Health Dis.* **1**(4), 208–215 (2010). 25141869 10.1017/S2040174410000115 PMC6691723

[4] Jungheim, E. S. & Moley, K. H. Current knowledge of obesity’s effects in the pre- and periconceptional periods and avenues for future research. *Am. J. Obstet. Gynecol.* **203**(6), 525–530 (2010). 20739012 10.1016/j.ajog.2010.06.043 PMC3718032

[5] Kovesdy, C. P., Furth, S. L. & Zoccali, C. Obesity and kidney disease: Hidden consequences of the epidemic. *Can. J. Kidney Health Dis.* **4**, 2054358117698669 (2017). 28540059 10.1177/2054358117698669 PMC5433675

[6] Australian Institute of Health Welfare. Chronic kidney disease (2020, AIHW: Canberra).

[7] Ejerblad, E. *et al.* Obesity and risk for chronic renal failure. *J. Am. Soc. Nephrol.* **17**(6), 1695–1702 (2006). 16641153 10.1681/ASN.2005060638

[8] Kramer, H. *et al.* Obesity and prevalent and incident CKD: The hypertension detection and follow-up program. *Am. J. Kidney Dis.* **46**(4), 587–594 (2005). 16183412 10.1053/j.ajkd.2005.06.007

[9] Chen, J. *et al.* The metabolic syndrome and chronic kidney disease in U.S. adults. *Ann. Intern. Med.* **140**(3), 167–174 (2004). 14757614 10.7326/0003-4819-140-3-200402030-00007

[10] Kahn, B. B. & Flier, J. S. Obesity and insulin resistance. *J. Clin. Investig.* **106**(4), 473–481 (2000). 10953022 10.1172/JCI10842 PMC380258

[11] Rodrigo, N., & Glastras, S. J. The emerging role of biomarkers in the diagnosis of gestational diabetes mellitus. *J. Clin. Med*. **7**(6) (2018). 10.3390/jcm7060120 PMC6024961 29882903

[12] Taylor, R. Insulin resistance and type 2 diabetes. *Diabetes* **61**(4), 778–779 (2012). 22442298 10.2337/db12-0073 PMC3314346

[13] Wisse, B. E. The inflammatory syndrome: The role of adipose tissue cytokines in metabolic disorders linked to obesity. *J. Am. Soc. Nephrol.* **15**(11), 2792–2800 (2004). 15504932 10.1097/01.ASN.0000141966.69934.21

[14] Locatelli, F., Pozzoni, P. & Del Vecchio, L. Renal manifestations in the metabolic syndrome. *J. Am. Soc. Nephrol.* **17**(4 suppl 2), S81 (2006). 16565254 10.1681/ASN.2005121332

[15] Wang, S. *et al.* Connective tissue growth factor in tubulointerstitial injury of diabetic nephropathy. *Kidney Int.* **60**(1), 96–105 (2001). 11422741 10.1046/j.1523-1755.2001.00776.x

[16] Straznicky, N. E. *et al.* Exercise augments weight loss induced improvement in renal function in obese metabolic syndrome individuals. *J. Hypertens.* **29**(3), 553–564 (2011). 21119532 10.1097/HJH.0b013e3283418875

[17] Zhao, X., *et al.* GLP-1 Receptor agonists: Beyond their pancreatic effects. Front. Endocrinol. **12** (2021). 10.3389/fendo.2021.721135 PMC8419463 34497589

[18] Russell-Jones, D. Molecular, pharmacological and clinical aspects of liraglutide, a once-daily human GLP-1 analogue. *Mol. Cell. Endocrinol.* **297**(1–2), 137–140 (2009). 19041364 10.1016/j.mce.2008.11.018

[19] Dailey, M. J. & Moran, T. H. Glucagon-like peptide 1 and appetite. *Trends Endocrinol. Metab.* **24**(2), 85–91 (2013). 23332584 10.1016/j.tem.2012.11.008 PMC3594872

[20] Glastras, S. J. *et al.* Effect of GLP-1 receptor activation on offspring kidney health in a rat model of maternal obesity. *Sci. Rep.* **6**, 23525–23525 (2016). 27004609 10.1038/srep23525 PMC4804207

[21] Rowlands, J., *et al.* Pleiotropic effects of GLP-1 and analogs on cell signaling, metabolism, and function. *Front. Endocrinol.*, 672 (2018). 10.3389/fendo.2018.00672 PMC6266510 30532733

[22] Greco, E. V. *et al.* GLP-1 receptor agonists and kidney protection. *Medicina* **55**(6), 233 (2019). 31159279 10.3390/medicina55060233 PMC6630923

[23] Skov, J. Effects of GLP-1 in the kidney. *Rev. Endocr. Metab. Disord.* **15**(3), 197–207 (2014). 24791975 10.1007/s11154-014-9287-7

[24] Kominiarek, M. A. & Chauhan, S. P. Obesity before, during, and after pregnancy: A review and comparison of five national guidelines. *Am. J. Perinatol.* **33**(5), 433–441 (2016). 26588260 10.1055/s-0035-1567856

[25] Kim, H. H. Preconception dilemma for women with obesity: Is it worth waiting to lose weight?. *Fertil. Steril.* **114**(6), 1175–1176 (2020). 33280725 10.1016/j.fertnstert.2020.10.033

[26] Rodrigo, N., *et al.* Preconception weight loss improves fertility and maternal outcomes in obese mice. *J. Endocrinol.*, JOE-21-0399 (2022). 10.1530/JOE-21-0399 35080198

[27] Candas, D. & Li, J. J. MnSOD in oxidative stress response-potential regulation via mitochondrial protein influx. *Antioxidants Redox Signal.* **20**(10), 1599–1617 (2014). 10.1089/ars.2013.5305 PMC3942709 23581847

[28] Liang, H. & Ward, W. F. PGC-1alpha: A key regulator of energy metabolism. *Adv. Physiol. Educ.* **30**(4), 145–151 (2006). 17108241 10.1152/advan.00052.2006

[29] Zandbergen, F. & Plutzky, J. PPARalpha in atherosclerosis and inflammation. *Biochim. Biophys. Acta* **1771**(8), 972–982 (2007). 17631413 10.1016/j.bbalip.2007.04.021 PMC2083576

[30] Cheng, C.-F., Chen, H.-H. & Lin, H. Role of PPARα and its agonist in renal diseases. *PPAR Res.* **2010**, 345098–345098 (2010). 21076544 10.1155/2010/345098 PMC2976496

[31] O'Brien, T. E., Ray, J. G., & Chan, W.-S. Maternal body mass index and the risk of preeclampsia: A systematic overview. *Epidemiology*, 368–374 (2003). 10.1097/00001648-200305000-00020 12859040

[32] Lewandowska, M., Więckowska, B. & Sajdak, S. Pre-pregnancy obesity, excessive gestational weight gain, and the risk of pregnancy-induced hypertension and gestational diabetes mellitus. *J. Clin. Med.* **9**(6), 1980 (2020). 32599847 10.3390/jcm9061980 PMC7355601

[33] Madan, J. *et al.* Maternal obesity, gestational hypertension, and preterm delivery. *J. Matern. Fetal Neonatal. Med.* **23**(1), 82–88 (2010). 19903115 10.3109/14767050903258738

[34] Williams, D. Long-term complications of preeclampsia. *Semin. Nephrol.* **31**(1), 111–122 (2011). 21266269 10.1016/j.semnephrol.2010.10.010

[35] Kovesdy, C. P. *et al.* Obesity and kidney disease: Hidden consequences of the epidemic. *Can. J. kidney Health Dis.* **4**, 2054358117698669–2054358117698669 (2017). 28540059 10.1177/2054358117698669 PMC5433675

[36] Dittmann, K. *et al.* U-shaped association between central body fat and the urinary albumin-to-creatinine ratio and microalbuminuria. *BMC Nephrol.* **14**(1), 87 (2013). 23594567 10.1186/1471-2369-14-87 PMC3637595

[37] Câmara, N. O. S. *et al.* Kidney disease and obesity: Epidemiology, mechanisms and treatment. *Nat. Rev. Nephrol.* **13**(3), 181–190 (2017). 28090083 10.1038/nrneph.2016.191

[38] Coward, R. & Fornoni, A. Insulin signaling: Implications for podocyte biology in diabetic kidney disease. *Curr. Opin. Nephrol. Hypertens.* **24**(1), 104–110 (2015). 25415617 10.1097/MNH.0000000000000078 PMC4386894

[39] Cheng, E. Obesity and inflammation: The fas connection. *Sci. Transl. Med.*, **2**(16), 16ec15–16ec15 (2010).

[40] Wagener, A. *et al.* Genetic and diet effects on Ppar-α and Ppar-γ signaling pathways in the Berlin Fat Mouse Inbred line with genetic predisposition for obesity. *Lipids Health Dis.* **9**(1), 99 (2010). 20831792 10.1186/1476-511X-9-99 PMC2944240

[41] Chambers, J. M. & Wingert, R. A. PGC-1α in disease: Recent renal insights into a versatile metabolic regulator. *Cells* **9**(10), 2234 (2020). 33022986 10.3390/cells9102234 PMC7601329

[42] García-Prieto, C. F. *et al.* High-fat diet induces endothelial dysfunction through a down-regulation of the endothelial AMPK–PI3K–Akt–eNOS pathway. *Mol. Nutr. Food Res.* **59**(3), 520–532 (2015). 25421217 10.1002/mnfr.201400539

[43] Sansbury, B. E. *et al.* Overexpression of endothelial nitric oxide synthase prevents diet-induced obesity and regulates adipocyte phenotype. *Circ. Res.* **111**(9), 1176–1189 (2012). 22896587 10.1161/CIRCRESAHA.112.266395 PMC3707504

[44] Huh, J. Y. *et al.* 8-Hydroxy-2-deoxyguanosine ameliorates high-fat diet-induced insulin resistance and adipocyte dysfunction in mice. *Biochem. Biophys. Res. Commun.* **491**(4), 890–896 (2017). 28754587 10.1016/j.bbrc.2017.07.132

[45] Kobayashi, M. *et al.* Contribution of PGC-1α to obesity- and caloric restriction-related physiological changes in white adipose tissue. *Int. J. Mol. Sci.* **22**(11), 6025 (2021). 34199596 10.3390/ijms22116025 PMC8199692

[46] Jensen-Urstad, A. P. & Semenkovich, C. F. Fatty acid synthase and liver triglyceride metabolism: Housekeeper or messenger?. *Biochim. Biophys. Acta* **1821**(5), 747–753 (2012). 22009142 10.1016/j.bbalip.2011.09.017 PMC3288544

[47] Bournat, J. C. & Brown, C. W. Mitochondrial dysfunction in obesity. *Curr. Opin. Endocrinol. Diabetes Obes.* **17**(5), 446–452 (2010). 20585248 10.1097/MED.0b013e32833c3026 PMC5001554

[48] Drucker, D. J. Mechanisms of action and therapeutic application of glucagon-like peptide-1. *Cell Metab.* **27**(4), 740–756 (2018). 29617641 10.1016/j.cmet.2018.03.001

[49] Petersen, K. E. *et al.* Does glucagon-like peptide-1 ameliorate oxidative stress in diabetes? Evidence based on experimental and clinical studies. *Curr. Diabetes Rev.* **12**(4), 331–358 (2016). 26381142 10.2174/1573399812666150918150608 PMC5101636

[50] Tran, K. L. *et al.* Overview of glucagon-like peptide-1 receptor agonists for the treatment of patients with type 2 diabetes. *Am. Health Drug Benefits* **10**(4), 178–188 (2017). 28794822 PMC5536194

[51] Aaboe, K. *et al.* GLP-1: Physiological effects and potential therapeutic applications. *Diabetes Obes. Metab.* **10**(11), 994–1003 (2008). 18435775 10.1111/j.1463-1326.2008.00853.x

[52] Hviid, A. V. R. & Sørensen, C. M. Glucagon-like peptide-1 receptors in the kidney: impact on renal autoregulation. *Am. J. Physiol. Renal Physiol.* **318**(2), F443–F454 (2019). 31841385 10.1152/ajprenal.00280.2019

[53] Hirata, K. *et al.* Exendin-4 has an anti-hypertensive effect in salt-sensitive mice model. *Biochem. Biophys. Res. Commun.* **380**(1), 44–49 (2009). 19150338 10.1016/j.bbrc.2009.01.003

[54] Elmarakby, A. A. & Sullivan, J. C. Relationship between oxidative stress and inflammatory cytokines in diabetic nephropathy. *Cardiovasc. Ther.* **30**(1), 49–59 (2012). 20718759 10.1111/j.1755-5922.2010.00218.x

[55] Fujita, H. *et al.* The protective roles of GLP-1R signaling in diabetic nephropathy: Possible mechanism and therapeutic potential. *Kidney Int.* **85**(3), 579–589 (2014). 24152968 10.1038/ki.2013.427

[56] Wang, C. *et al.* GLP-1 receptor agonist ameliorates obesity-induced chronic kidney injury via restoring renal metabolism homeostasis. *PLoS ONE* **13**(3), e0193473 (2018). 29590132 10.1371/journal.pone.0193473 PMC5873987

[57] Muller, D. R. P., *et al.* Effects of GLP-1 agonists and SGLT2 inhibitors during pregnancy and lactation on offspring outcomes: A systematic review of the evidence. *Front. Endocrinol*. **14** (2023). 10.3389/fendo.2023.1215356 PMC10597691 37881498

[58] Gæde, P. *et al.* Effect of a multifactorial intervention on mortality in type 2 diabetes. *N. Engl. J. Med.* **358**(6), 580–591 (2008). 18256393 10.1056/NEJMoa0706245

[59] Group & U.P.D.S.,. Intensive blood-glucose control with sulphonylureas or insulin compared with conventional treatment and risk of complications in patients with type 2 diabetes (UKPDS 33). *The Lancet* **352**(9131), 837–853 (1998). 10.1016/S0140-6736(98)07019-6 9742976

[60] Control, D. and C.T.R. Group. The effect of intensive treatment of diabetes on the development and progression of long-term complications in insulin-dependent diabetes mellitus. *N. Engl. J. Med.* **329**(14), 977–986 (1993). 8366922 10.1056/NEJM199309303291401

[61] Viñas Esmel, E., Naval Álvarez, J. & Sacanella Meseguer, E. The legacy effect in the prevention of cardiovascular disease. *Nutrients* **12**(11), 3227 (2020). 33105611 10.3390/nu12113227 PMC7690390

[62] de Cabo, R. & Mattson, M. P. Effects of intermittent fasting on health, aging, and disease. *N. Engl. J. Med.* **381**(26), 2541–2551 (2019). 31881139 10.1056/NEJMra1905136

[63] Glastras, S. J. *et al.* Mouse Models of diabetes, obesity and related kidney disease. *PLoS ONE* **11**(8), e0162131 (2016). 27579698 10.1371/journal.pone.0162131 PMC5006968

[64] Fransson, L. *et al.* Liraglutide counteracts obesity and glucose intolerance in a mouse model of glucocorticoid-induced metabolic syndrome. *Diabetol. Metab. Syndrome* **6**(1), 3 (2014). 10.1186/1758-5996-6-3 PMC3905931 24423471

